# Recurrent Pregnancy Loss Despite Therapeutic Anticoagulation in a Patient With May-Thurner Syndrome: A Case Report

**DOI:** 10.7759/cureus.107577

**Published:** 2026-04-23

**Authors:** Fiona Lin, Adib Khan, Hibah Mohammed, Tanner F Wise, Hussain Rawiji

**Affiliations:** 1 Medicine, Lake Erie College of Osteopathic Medicine (LECOM) - Bradenton, Bradenton, USA; 2 Pediatrics and Obstetrics, AdventHealth Central Florida, Orange City, USA

**Keywords:** deep vein thrombosis, fetal demise, may-thurner syndrome, placental insufficiency, pregnancy, venous outflow obstruction

## Abstract

Pregnancy is a hypercoagulable state associated with an increased risk of venous thromboembolism. May-Thurner syndrome (MTS), an anatomic compression of the left common iliac vein by the right common iliac artery, predisposes affected individuals to left-sided deep vein thrombosis (DVT) and is often underrecognized in pregnant patients. While maternal thrombotic complications related to MTS have been described, its potential impact on fetal outcomes remains poorly characterized. We report the case of a 29-year-old gravida 7, para 2 woman with confirmed MTS and recurrent pregnancy-associated left lower extremity DVTs who experienced multiple adverse obstetric outcomes, including fetal growth restriction and recurrent fetal demise. Despite negative hypercoagulable testing and adherence to therapeutic anticoagulation, the patient developed postpartum and early-pregnancy DVTs and demonstrated a progressive pattern of fetal loss at increasingly earlier gestational ages. Her most recent pregnancy ended in early second-trimester fetal demise while receiving therapeutic low-molecular-weight heparin. This case suggests a clinically relevant association between maternal venous outflow obstruction, recurrent thrombosis, and placental dysfunction. The combination of chronic iliac venous compression and recurrent thromboembolic events may impair uteroplacental venous drainage, contributing to placental insufficiency, fetal growth restriction, and fetal demise. Notably, these outcomes occurred in the absence of systemic thrombophilia, suggesting a predominantly anatomic and physiologic mechanism.

## Introduction

Pregnancy is a physiologic hypercoagulable state characterized by increased clotting factors, reduced fibrinolytic activity, and venous stasis, all of which contribute to an elevated risk of venous thromboembolism (VTE) [1,2]. VTE occurs in 1-2 per 1000 pregnancies, with pregnancy conferring a four to fivefold increased risk compared to the non-pregnant state [1,3]. Deep vein thrombosis (DVT) remains a major cause of maternal morbidity, and anticoagulation is the cornerstone of management [3]. However, recurrent thrombotic events and adverse pregnancy outcomes may still occur despite appropriate therapy, suggesting that factors beyond systemic hypercoagulability may be involved.

May-Thurner syndrome (MTS) is a vascular condition in which the right common iliac artery compresses the left common iliac vein against the lumbar spine, resulting in chronic venous outflow obstruction [4,5]. This anatomic variant accounts for 2-5% of all DVTs and predisposes patients to left-sided lower extremity DVT, frequently affecting young women in the peripartum period [4]. In pregnancy, recognition of MTS is particularly challenging, as physiologic venous compression and pregnancy-related symptoms may obscure the diagnosis. The left-sided predominance of pregnancy-associated DVT (85% vs. 55% in non-pregnant women) has been attributed to May-Thurner anatomy coupled with compression of the vena cava by the gravid uterus [3,6].

Prior literature on MTS in pregnancy mainly focuses on maternal thrombotic complications, with limited attention to obstetric or fetal outcomes [7]. There are no current standardized management guidelines, and the potential impact of maternal venous outflow obstruction on placental perfusion remains poorly defined. We present a case of recurrent pregnancy-associated DVT due to MTS complicated by fetal growth restriction and fetal demise despite therapeutic anticoagulation, highlighting a potential link between maternal venous pathology and adverse fetal outcomes.

## Case presentation

We present a 29-year-old gravida 7, para 2 woman with a history of MTS and recurrent DVTs who presented to labor and delivery triage with decreased fetal movement and was found to have early second-trimester fetal demise. She has a past medical history of hydrocephalus and a ventriculoperitoneal shunt since childhood.

The patient’s first pregnancy resulted in the spontaneous vaginal delivery of a full-term male infant who was later diagnosed with epilepsy secondary to an in utero stroke. This pregnancy was notable for inadequate prenatal care, including Group B Streptococcus screening. Her second pregnancy progressed to 36 weeks and 0 days of gestation and ended due to placental abruption. The third pregnancy resulted in the delivery of a healthy full-term male infant. A dilation and curettage procedure was performed postpartum due to a retained placenta. In February 2021, while having symptoms of nausea and vomiting, she delivered a one-pound, one-ounce nonviable fetus at home, not knowing she was pregnant.

In May 2023, the patient had her fifth pregnancy at 20 weeks and three days of gestation. Anatomy ultrasound demonstrated fetal hydrops. Fetal examination revealed a possible cystic hygroma on the posterior inferior neck and a possible right-sided cleft lip. The fetus appeared macerated with skin sloughing and exhibited global edema.

In August 2024, the patient presented as gravida 6 at approximately 25 weeks of gestation to the emergency department with tingling involving both sides of her face as well as her hands and feet. These symptoms resolved approximately two hours before arrival. Laboratory evaluation revealed hypokalemia with a potassium level of 2.0 mmol/L, and she was treated with intravenous potassium infusion. Obstetrics was consulted, and fetal heart tones were reassuring. At 33 weeks and three days of gestation during this sixth pregnancy, she underwent ultrasound evaluation and maternal-fetal medicine consultation. The estimated fetal weight was 1,841 grams, corresponding to the seventh percentile for gestational age, consistent with fetal growth restriction. Femur length measured at the first percentile; however, fetal morphology appeared normal. The biophysical profile score was 8 out of 8, and the patient reported good fetal movement.

In November 2024, at 34 weeks and 0 days of gestation, the patient was admitted for inpatient management of fetal growth restriction in the setting of a poor obstetric history. Continuous fetal monitoring demonstrated prolonged fetal heart rate decelerations, and maternal-fetal medicine recommended cesarean delivery. A female infant weighing 3 pounds 11.6 ounces was delivered, with Appearance, Pulse, Grimace, Activity, and Respiration (APGAR) scores of 7 and 9 at one and five minutes, respectively.

**Figure 1:**
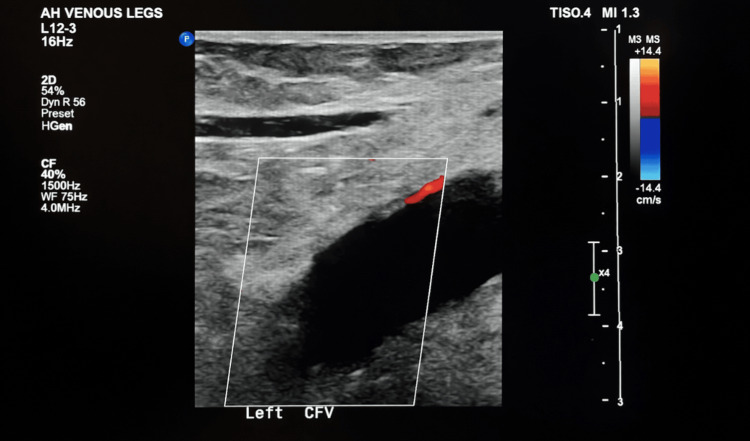
Venous Doppler ultrasound depicting extensive left lower extremity DVT with occlusive thrombus extending from the common femoral vein to the trifurcation. The red Doppler signal means venous blood towards the transducer, consistent with occlusive DVT. DVT: deep vein thrombosis

Approximately two weeks later, while taking aspirin 81 mg three times daily as told by obstetrics, the patient presented to the emergency department with left lower extremity pain and swelling described as throbbing and aching diffusely in the left thigh, along with discoloration. She also reported mild shortness of breath and denied chest pain. Three days later, Doppler ultrasound demonstrated extensive left lower extremity DVT with occlusive thrombus extending from the common femoral vein (Figure 1). Hematology-oncology was consulted, and computed tomography venography of the abdomen and pelvis demonstrated anatomy consistent with MTS, in which the left common iliac vein was compressed as it crosses the L4-5 vertebral body. The patient underwent thrombectomy with common iliac vein stent placement performed by interventional radiology. She also received multiple iron infusions for iron-deficiency anemia and was cleared for outpatient anticoagulation. She was discharged on apixaban 10 mg twice daily for seven days, followed by 5 mg twice daily. Interventional radiology recommended continuation of low-dose aspirin for six months.

In May 2025, the patient presented to the emergency department with her seventh pregnancy. She reported that anticoagulation had been discontinued approximately two months earlier following a negative hypercoagulable workup. Doppler ultrasound revealed an acute left lower extremity DVT involving the common femoral, femoral, and popliteal veins. She was also incidentally found to be in early pregnancy. Interventional radiology did not recommend thrombectomy due to pregnancy, and the patient was admitted for further evaluation. She was started on a heparin drip. Hematology-oncology and obstetrics-gynecology were consulted, and both services recommended transition to enoxaparin 1.5 mg/kg once daily for outpatient management with close follow-up. The patient understood and agreed with the treatment plan.

**Figure 2:**
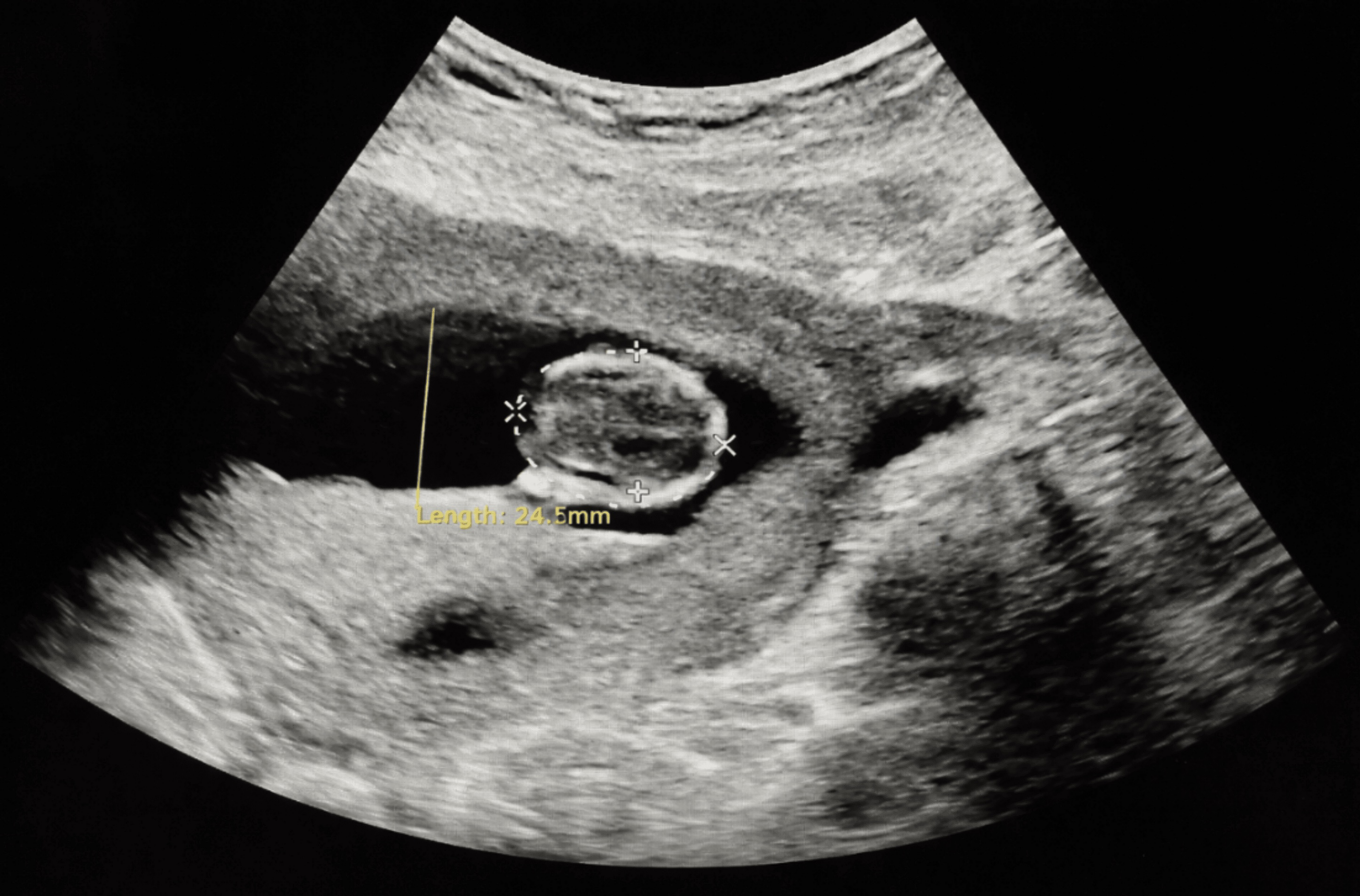
Transabdominal ultrasound depicting a fetus in the patient’s cervix at 13 weeks and five days of gestation.

In July 2025, the patient presented to the obstetric triage with a chief complaint of decreased fetal movement. She was taking enoxaparin 60 mg/0.6 mL via prefilled syringe, administering 0.6 mL (60 mg) subcutaneously every 12 hours. Laboratory evaluation, including prothrombin time, activated partial thromboplastin time, international normalized ratio, and fibrinogen levels, was within normal limits (Table 1). She subsequently experienced spontaneous delivery of a nonviable fetus at 13 weeks and five days of gestation (Figure 2). APGAR scores were 0 at both one and five minutes.

**Table 1:** Normal coagulation studies at 13 weeks and five days of gestation ultrasound.

Parameter (Units)	Lab Value	Reference Range
Prothrombin time (seconds)	13.0	11.5-14.9
International normalized ratio	0.97	<3.5
Activated partial thromboplastin time (seconds)	25.3	22.4-38.6
Fibrinogen (mg/dL)	383	200-500

## Discussion

This case describes a patient with confirmed MTS who experienced recurrent pregnancy-associated left-sided DVTs and adverse fetal outcomes, including fetal growth restriction and fetal demise. A notable feature of this case is the progression toward earlier gestational fetal loss with recurrent thromboembolic events. Although causation cannot be established, the observed temporal pattern raises concern for a cumulative effect of maternal venous pathology on pregnancy outcomes.

MTS results from chronic compression of the left common iliac vein, leading to venous stasis and endothelial injury [4,5]. Pregnancy exacerbates these effects through increased circulating blood volume and mechanical compression from the gravid uterus, creating a localized prothrombotic environment even in the absence of systemic coagulation abnormalities [2,8]. This localized prothrombotic environment may help explain recurrent thrombosis in this patient despite normal coagulation studies and a negative hypercoagulable evaluation. Recent registry data demonstrate that MTS is associated with a 2.26-fold increased risk of recurrent DVT despite aggressive anticoagulation therapy [9].

Beyond maternal thrombosis, chronic venous outflow obstruction and recurrent DVTs may impair uteroplacental venous drainage, contributing to placental hypoperfusion. While the literature on direct effects of maternal DVT on placental perfusion is limited, one case report documented transient fetal hypoxia with abnormal umbilical and cerebral Doppler indices during maternal iliofemoral DVT, with normalization following anticoagulation [10]. Women with a history of VTE have demonstrated increased risk of placenta-mediated complications, including preeclampsia (adjusted relative risk (RR) 1.5), stillbirth (adjusted RR 1.8), and placental abruption (adjusted RR 1.6) [11]. Placental congestion and impaired venous return may contribute to placental insufficiency, fetal growth restriction, and fetal demise. In this case, MTS likely provided the anatomic substrate, while recurrent thrombotic events amplified placental compromise. This relationship remains underreported in the literature.

The patient's normal coagulation parameters, adherence to anticoagulation, and negative hypercoagulable workup support an anatomic-physiologic mechanism rather than systemic thrombophilia. Clinically, this case suggests that recurrent DVTs in pregnancy, particularly when left-sided or refractory to standard therapy, may warrant heightened fetal surveillance and consideration of underlying venous pathology such as MTS.

## Conclusions

This case highlights MTS as a potentially underrecognized contributor to adverse fetal outcomes in pregnant patients with recurrent DVT. The observed association between maternal venous outflow obstruction, recurrent thrombosis, and progressively earlier fetal demise underscores the need for further investigation into the role of maternal venous disease in placental dysfunction. Increased recognition of this entity may inform risk stratification and management strategies in high-risk pregnancies.
